# Sonographic Features of Triple-Negative Breast Carcinomas Are Correlated With mRNA–lncRNA Signatures and Risk of Tumor Recurrence

**DOI:** 10.3389/fonc.2020.587422

**Published:** 2021-01-19

**Authors:** Jia-wei Li, Jin Zhou, Zhao-ting Shi, Na Li, Shi-chong Zhou, Cai Chang

**Affiliations:** ^1^ Department of Medical Ultrasound, Fudan University Shanghai Cancer Center, Shanghai, China; ^2^ Department of Oncology, Shanghai Medical College, Fudan University, Shanghai, China

**Keywords:** long non-coding RNA, messenger RNA, triple-negative breast cancer, tumor recurrence, ultrasound

## Abstract

**Background:**

To determine a correlation between mRNA and lncRNA signatures, sonographic features, and risk of recurrence in triple-negative breast cancers (TNBC).

**Methods:**

We retrospectively reviewed the data from 114 TNBC patients having undergone transcriptome analysis. The risk of tumor recurrence was determined based on the correlation between transcriptome profiles and recurrence-free survival. Ultrasound (US) features were described according to the Breast Imaging Reporting and Data System. Multivariate logistic regression analysis determined the correlation between US features and risk of recurrence. The predictive value of sonographic features in determining tumor recurrence was analyzed using receiver operating characteristic curves.

**Results:**

Three mRNAs (CHRDL1, FCGR1A, and RSAD2) and two lncRNAs (HIF1A-AS2 and AK124454) were correlated with recurrence-free survival in patients with TNBC. Among the three mRNAs, two were upregulated (FCGR1A and RSAD2) and one was downregulated (CHRDL1) in TNBCs. LncRNAs HIF1A-AS2 and AK124454 were upregulated in TNBCs. Based on these signatures, an integrated mRNA–lncRNA model was established using Cox regression analysis to determine the risk of tumor recurrence. Benign-like sonographic features, such as regular shape, circumscribed margin, posterior acoustic enhancement, and no calcifications, were associated with HIF1A-AS2 expression and high risk of tumor recurrence (P<0.05). Malignant-like features, such as irregular shape, uncircumscribed margin, no posterior acoustic enhancement, and calcifications, were correlated with CHRDL1 expression and low risk of tumor recurrence (P<0.05).

**Conclusions:**

Sonographic features and mRNA–lncRNA signatures in TNBCs represent the risk of tumor recurrence. Taken together, US may be a promising technique in determining the prognosis of patients with TNBC.

## Highlights

1. Sonographic features of TNBCs correlated with the expression of mRNAs and lncRNAs.
2. Benign-like sonographic features correlated with the expression of lncRNA HIF1A-AS2; malignant-like sonographic features correlated with CHRDL1 mRNA levels.
3. Sonographic features of TNBCs correlated with the risk of tumor recurrence predicted using the mRNA and lncRNA signatures.
4. TNBCs with benign-like sonographic features exhibited higher rate of recurrence than those with malignant sonographic features.

## Background

Triple-negative breast cancer (TNBC) is the most aggressive breast cancer subtype. Patients with TNBC lack the expression of estrogen receptor (ER), progesterone receptor (PR), and human epidermal growth factor receptor 2 (HER2). The aggressive nature of the cancer and limited availability of effective targeted therapy against molecular biomarkers result in poor prognosis of patients with TNBC (1).

Numerous studies have analyzed the genome and transcriptome signatures to identify the therapeutic target for TNBCs (2–6). TNBC heterogeneity has gained considerable attention in understanding therapeutic strategies and clinical outcomes (2–4, 7). Using the largest TNBC database at Fudan University Shanghai Cancer Center, a recent study confirmed the importance of personalized therapy based on the gene expression profile in patients with TNBC (3). They found that PIK3CA mutations and LAR subtype are more common in Chinese TNBC patients. Substantial evidence was established for the biological heterogeneity of TNBCs based on the genomic and transcriptomic profiles (3).

In accordance with TNBC heterogeneity, we have previously demonstrated that these tumors present with a wide variety of sonographic features (8) that correlate with cancer grade and score based on immunohistochemical (IHC) biomarkers, such as Ki-67 and HER2. However, these protein biomarkers have limited significance in the sonographic variations. In this study, we analyzed the sonographic variations based on messenger RNA (mRNA) and long non-coding RNA (lncRNA) signatures. Moreover, we have determined a correlation between sonographic features and risk of tumor recurrence.

## Methods

### Patients

Based on a prospective study between 1^st^ January 2010 and 31^st^ December 2013, 165 consecutive breast cancer patients having undergone transcriptome analysis were retrospectively reviewed for their clinical data and ultrasound (US) images. All patients were primarily treated by surgery after excluding surgical contraindications based on standard preoperative examination. None of the patients expressed ER, PR, and HER2 (characteristic of TNBC).

### Transcriptome Microarray, Identifying Candidate RNAs, and Integrated mRNA–lncRNA Model

A study in 2016 described the correlation between mRNAs and lncRNAs with recurrence-free survival (RFS) and established an integrated mRNA–lncRNA model to predict the risk of recurrence (2).

Differentially expressed RNAs were first identified from 33 paired TNBC tissues and adjacent normal breast tissues using HTA 2.0 microarray analysis. The inclusion criteria for these mRNAs included: fold change >2 or <0.33 and false discovery rate (FDR) <0.001. For lncRNAs, the inclusion criteria were: fold change >1.5 and FDR <0.001. Based on this, there were 183 and 195 differentially expressed mRNAs and lncRNAs, respectively, between TNBC and normal breast tissues. Combining the RNA expression data from a microarray with the clinical follow-up data from 165 TNBC patients, 16 mRNAs (P<0.1) and 11 lncRNAs (P<0.2) correlated with RFS using the log-rank test. Duplicated mRNAs were excluded and only intergenic lncRNAs were included and 13 mRNAs and 6 lncRNAs were identified using real-time quantitative polymerase chain reaction from 33 paired TNBC and normal tissues. Nineteen RNAs were consistently amplified in 137 training samples undergoing real-time quantitative polymerase chain reaction using the correlation with survival analysis as the filtration standard. The expression of RNA candidates that did not correlate with RFS were excluded. After this round of filtration, the remaining seven mRNAs and four lncRNAs were tested for their prognostic signature until the area under curve (AUC) in the time-dependent receiver operating characteristic model arrived at the best performance. Finally, there were three mRNAs (CHRDL1, FCGR1A, and RSAD2) and two lncRNAs (HIF1A-AS2 and AK124454) in the model (derived and rephrased from the **Supplementary File** of Jiang et al., 2016) (2). Of the three mRNAs, two were upregulated (FCGR1A and RSAD2) and one was downregulated (CHRDL1) during the progression of TNBC. LncRNAs HIF1A-AS2 and AK124454 were upregulated during TNBC progression.

The signature model for predicting the risk of recurrence was established with Cox proportional hazards regression modeling as: −1.225×CHRDL1+0.74×FCGR1A+0.219×RSAD2+0.482×HIF1A-AS2+0.571×AK124454. The cut-off score was set to 0.793 to classify patients into high-risk and low-risk groups (2).

### Pathological and Immunohistochemical Data

After measuring the gross tumor size, postoperative breast cancer samples were subjected to hematoxylin-eosin staining and IHC analysis. Pathological type, histological grade (I, II, and III), and lymph node status were determined based on the stained samples. Based on IHC analysis and *in situ* hybridization, the expression of ER, PR, and HER2 was confirmed by two pathologists with more than 10 years of working experiences. ER and PR negative expression was defined as less than 1% staining in nuclei. HER2 negative expression was defined by score 0 or 1 by IHC staining and <4 gene copies/nucleus and ratio of <1.8 using fluorescence *in situ* hybridization (9). Thus, TNBCs are defined as ER, PR, and HER2 negative (10). Ki-67 expression was also acquired from the IHC samples.

### Sonographic Feature Assessment

US images were retrospectively collected from the data archives. Fifty-one patients without US images were excluded. Images from 114 patients were blind reviewed by two US physicians with more than five years’ experience on breast US. TNBC masses were assessed for sonographic features based on orientation (parallel/non-parallel), shape (regular/irregular), margin (circumscribed/uncircumscribed), angular or spiculated margin (yes/no), echo pattern (hypoechoic, mixed solid echo, complex cystic and solid echo), posterior acoustic pattern (shadow, enhancement, no change, or mixed pattern), and calcification (yes/no). These sonographic features were based on the Breast Imaging Reporting and Data System published in 2013 (8, 11, 12). A final assessment report was established after incorporating the reviews by two US physicians.

According to our previous study (8), typical malignant sonographic features for TNBCs included irregular shape, angular or spiculated margin, posterior acoustic shadow, and presence of calcification. TNBCs were divided into three groups based on the number of sonographic features in the US images of the malignancies: Group 1, no malignant sonographic features; Group 2, one or two malignant sonographic features; and Group 3, three or four malignant sonographic features (13).

All data included in our study were ethically approved by the institutional review board at Fudan University Shanghai Cancer Center. The approval number for the data from the breast cancer microarray and clusters was 050432-4-1212B; all the patients provided written informed consent. The approval number for the review of data from the US images was 1802181-22-NSFC; due to the retrospective design of the study, patient written consent was waived.

### Statistical Analysis

Statistical analysis was performed using SPSS version 22.0 for Windows (SPSS Inc., Chicago, IL, USA) and MedCalc Statistical Software version 19.0.7 (MedCalc Software bvba, Ostend, Belgium). Continuous numerical variables were compared using the independent samples t-test, while categorical data were compared by Pearson’s chi-square test using a two-tailed P value <0.05 as statistically significant. Univariate and multivariate logistic regression analyses were used to determine the sonographic features associated with the risk of recurrence. Odds ratio (OR) with 95% confidence interval (CI) was calculated to identify the correlation between sonographic features and risk of recurrence. The prediction model for the risk of recurrence was established based on sonographic features and clinicopathological characteristics of patients. Receiver operating characteristic curves were used to determine the performance of the prediction model. Sensitivity, specificity, and AUC with 95% CI were calculated.

## Results

**Figure 1:**
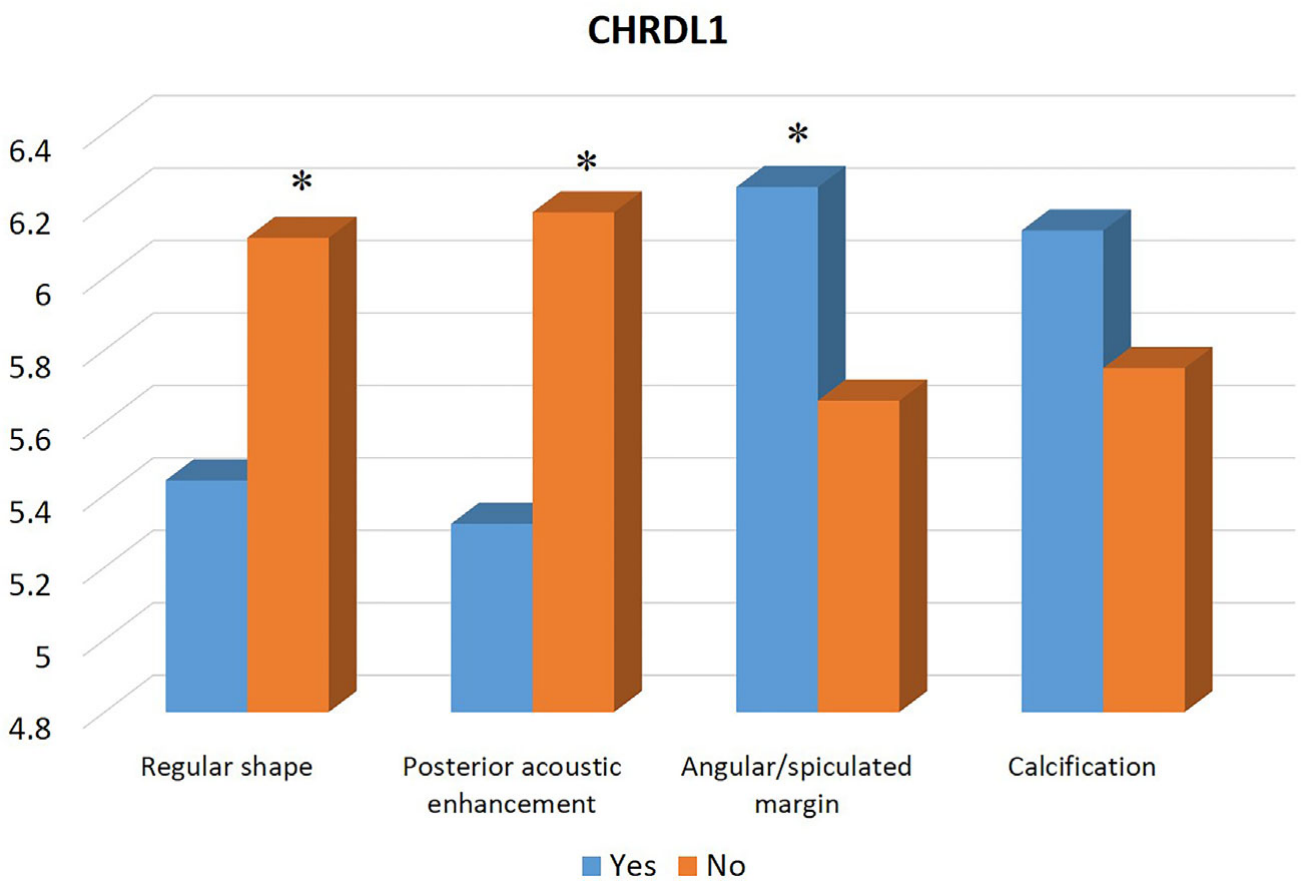
Association between the expression profile of mRNA CHRDL1 and sonographic features of TNBCs. The high expression of CHRDL1 is significantly associated with malignant sonographic features of irregular shape, absence of posterior acoustic enhancement, and presence of angular/spiculated margin. * indicates significant difference between the two groups with and without the specific sonographic feature.

**Figure 2:**
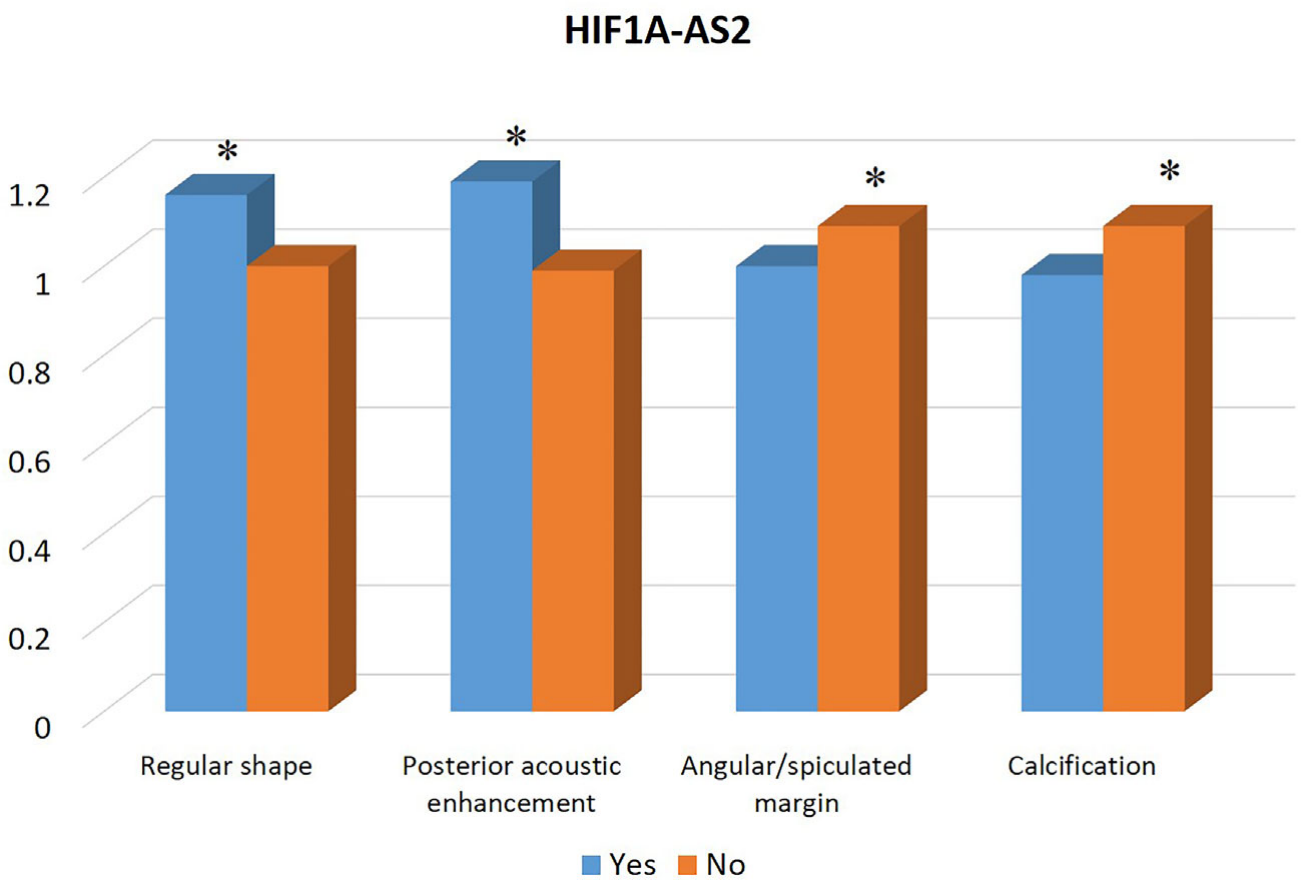
Association between the expression profile of lncRNA HIF1A-AS2 and sonographic features of TNBCs. The high expression of HIF1A-AS2 is significantly associated with benign sonographic features of regular shape, presence of posterior acoustic enhancement, absence of angular/spiculated margin, and no calcification. * indicates significant difference between the two groups with and without the specific sonographic feature.

**Figure 3:**
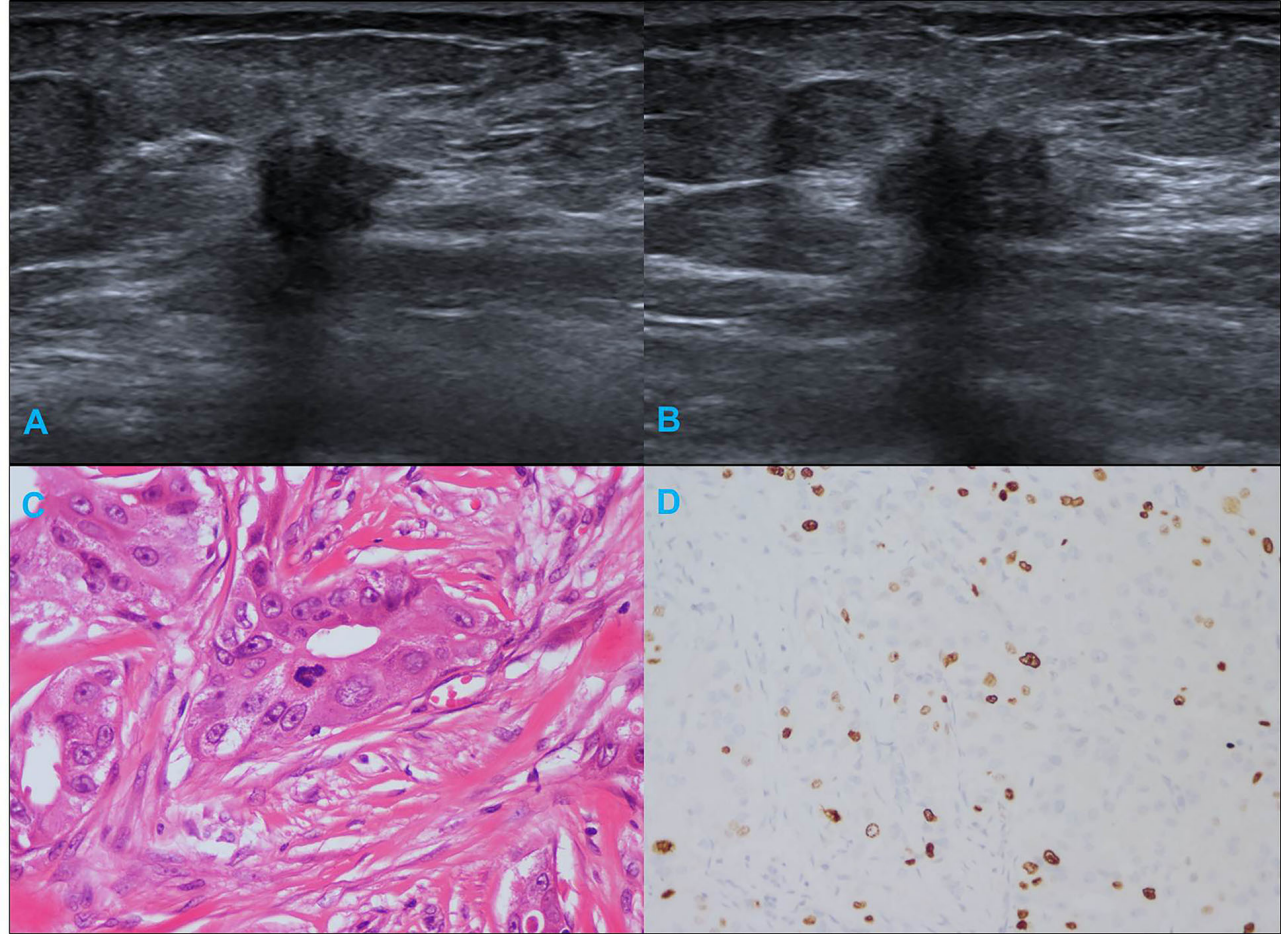
Illustration of TNBC in a 66-year-old female patient. **(A, B)** Irregular shape, angular margin and posterior acoustic shadow in sonogram (BI-RADS:4C); **(C)** Histological grade II in HE staining (original magnification × 400); **(D)** Twenty percent Ki-67 expression in IHC staining (original magnification × 200).

**Figure 4:**
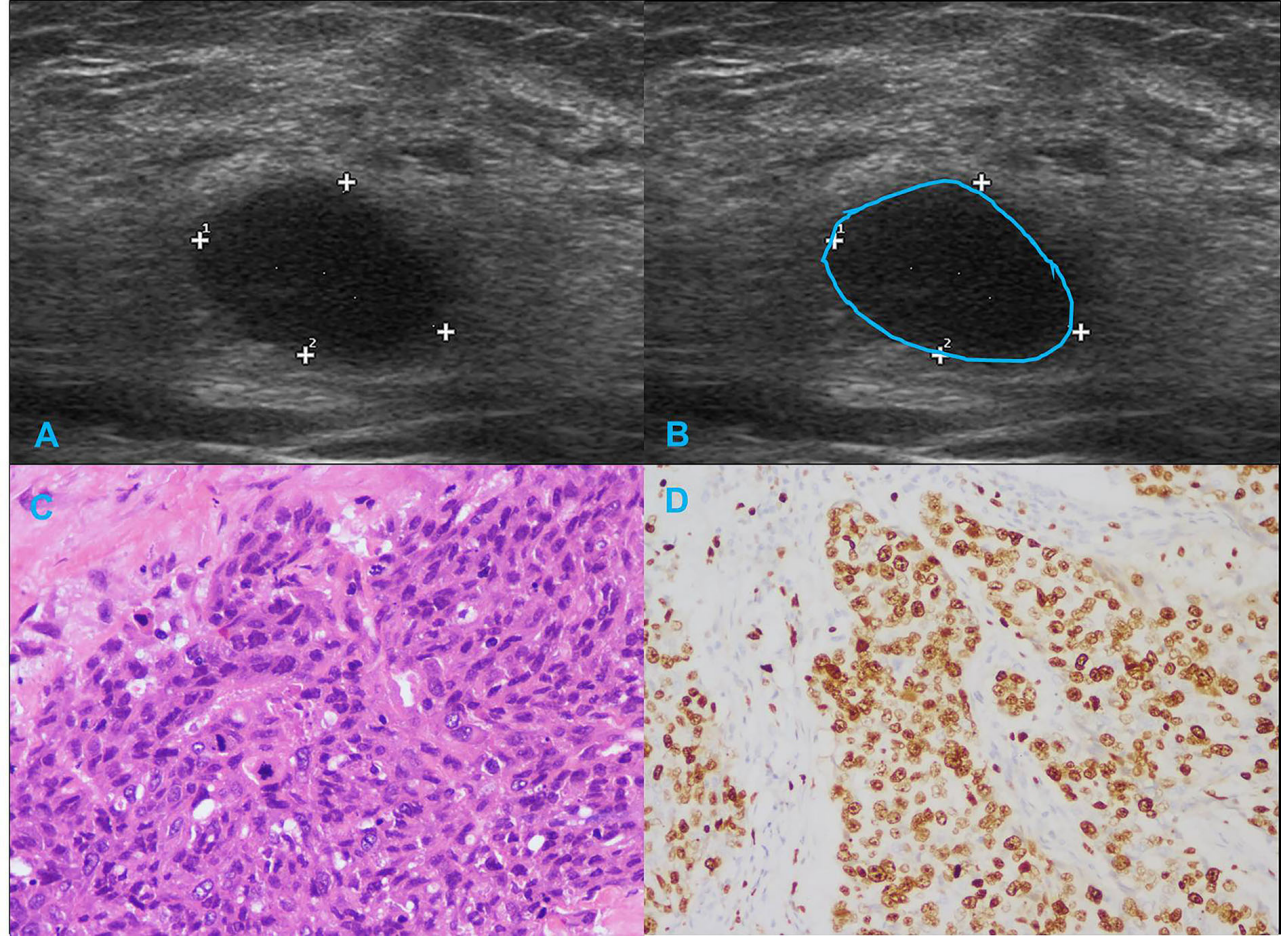
Illustration of TNBC in a 29-year-old female patient. **(A, B)** Regular shape, circumscribed margin and posterior acoustic enhancement in sonogram (BI-RADS:4A); **(C)** Histological grade III in HE staining (original magnification × 400); **(D)** Eighty percent Ki-67 expression in IHC staining (original magnification × 200).

**Table 1** summarizes the clinicopathological and IHC data from the 114 patients. **Table 2** summarizes the expression profiles of five RNAs categorized by sonographic features. Malignant-like sonographic features, such as irregular shape, uncircumscribed margin, presence of angular/spiculated margin, and absence of posterior acoustic enhancement, were associated with high expression of CHRDL1 (P<0.05, **Figure 1**). Benign-like sonographic features, such as regular shape, no angular/spiculated margin, posterior acoustic enhancement, and no calcification, positively associated with HIF1A-AS2 (P ≤ 0.05, **Figure 2**). **Figure 3** illustrates TNBC masses with malignant-like sonographic appearances with low rates of cellular proliferation. **Figure 4** illustrates TNBC masses with benign-like sonographic appearances with high cellular proliferation.

**Table 1:** Clinicopathological and IHC characteristics of all TNBC patients.

Age (yrs)	53.4 ± 9.7	Tumor size (cm)	2.44 ± 0.84
**Surgical type**		**Pathological type**	
MRM	82 (71.9%)	IDC	104 (91.2%)
M+SLNB	32 (28.1%)	DCIS	2 (1.8%)
**Chemotherapy**		ILC	2 (1.8%)
Taxane-based	87 (76.3%)	Others	6 (5.3%)
Non-taxane-based	18 (15.8%)	**Histological grade**	
Unknown	9 (7.9%)	I and II	38 (33.3%)
**Radiotherapy**		III	76 (66.7%)
Yes	31 (27.2%)	**LNM**	
No	73 (64.0%)	Yes	35 (30.7%)
Unknown	10 (8.8%)	No	79 (69.3%)
**Follow up (month)**	63 (57–70)	**Ki-67 (%)**	60 (30–70)

Categorical data are presented as number (%). Numerical data are presented as mean ± SD or median (IQR).

DCIS, Ductal carcinoma in situ; IDC, Infiltrative ductal carcinoma; ILC, Infiltrative lobular carcinoma; IQR, Interquartile range; LNM, Lymph node metastasis; M, Mastectomy; MRM, Modified radical mastectomy; SLNB, Sentinel lymph node biopsy.

**Table 2:** Expression level of mRNAs and lncRNAs in associated with sonographic features of TNBC patients.

	CHRDL1	FCGR1A	RSAD2	HIF1A-AS2	AK124454
**Shape**					
Regular	5.44 ± 1.66	4.94±0.77	5.84±1.00	1.16 ± 0.36	3.58±0.97
Irregular	6.11 ± 1.55	4.74±0.75	5.65±1.17	1.00±0.18	3.53±0.84
**P value**	0.031*	0.176	0.382	0.011*	0.778
**Margin**					
Circumscribed	4.73 ± 1.60	5.30 ± 0.76	5.77 ± 0.82	1.07±0.24	3.44±0.75
Uncircumscribed	5.99 ± 1.58	4.76 ± 0.74	5.71±1.14	1.06±0.28	3.56±0.90
**P value**	0.01*	0.019*	0.873	0.893	0.66
**Angular margin**					
Yes	6.25 ± 1.44	4.76±0.77	5.63±1.06	1.00 ± 0.18	3.58±0.90
No	5.66 ± 1.68	4.84±0.76	5.77±1.13	1.09 ± 0.31	3.54±0.88
**P value**	0.049*	0.601	0.531	0.05*	0.786
**Echo pattern**					
Hypoechoic	5.81 ± 1.54	5.01 ± 0.74	5.99 ± 1.06	1.10±0.33	3.55±0.79
Mixed	6.00 ± 1.64	4.60 ± 0.73	5.48 ± 1.10	1.03±0.21	3.56±0.98
Solid and cystic	3.05 ± 0.12	5.70 ± 0.20	4.92 ± 0.62	1.15±0.32	3.43±1.04
**P value**	0.038*	0.003*	0.029*	0.377	0.980
**Posterior acoustic pattern**					
Shadow	6.57 ± 1.18	4.88±0.99	5.75±1.38	0.96 ± 0.13	3.51±1.07
Enhancement	5.32 ± 1.70	4.97±0.76	5.88±1.09	1.19 ± 0.36	3.56±0.88
No change	6.08 ± 1.51	4.71±0.72	5.59±1.06	0.98 ± 0.16	3.55±0.90
Mixed	6.44 ± 1.65	4.70±0.79	5.67±1.26	1.05 ± 0.22	3.54±0.83
**P value**	0.033*	0.366	0.642	0.001*	0.999
**Posterior acoustic enhancement**					
Yes	5.32 ± 1.70	4.97±0.76	5.88±1.09	1.19 ± 0.36	3.56±0.88
No	6.18 ± 1.49	4.72±0.75	5.62±1.11	0.99 ± 0.17	3.55±0.90
**P value**	0.005*	0.094	0.216	0.001*	0.940
**Calcification**					
Yes	6.13±1.60	4.68±0.68	5.71±1.10	0.98 ± 0.13	3.56±0.83
No	5.75±1.62	4.87±0.79	5.72±1.12	1.09 ± 0.31	3.52±1.03
**P value**	0.267	0.243	0.958	0.01*	0.82

Data are presented as mean ± SD for RNAs. * Indicates significant difference. Bold values indicate P values less than 0.05.

**Figure 5:**
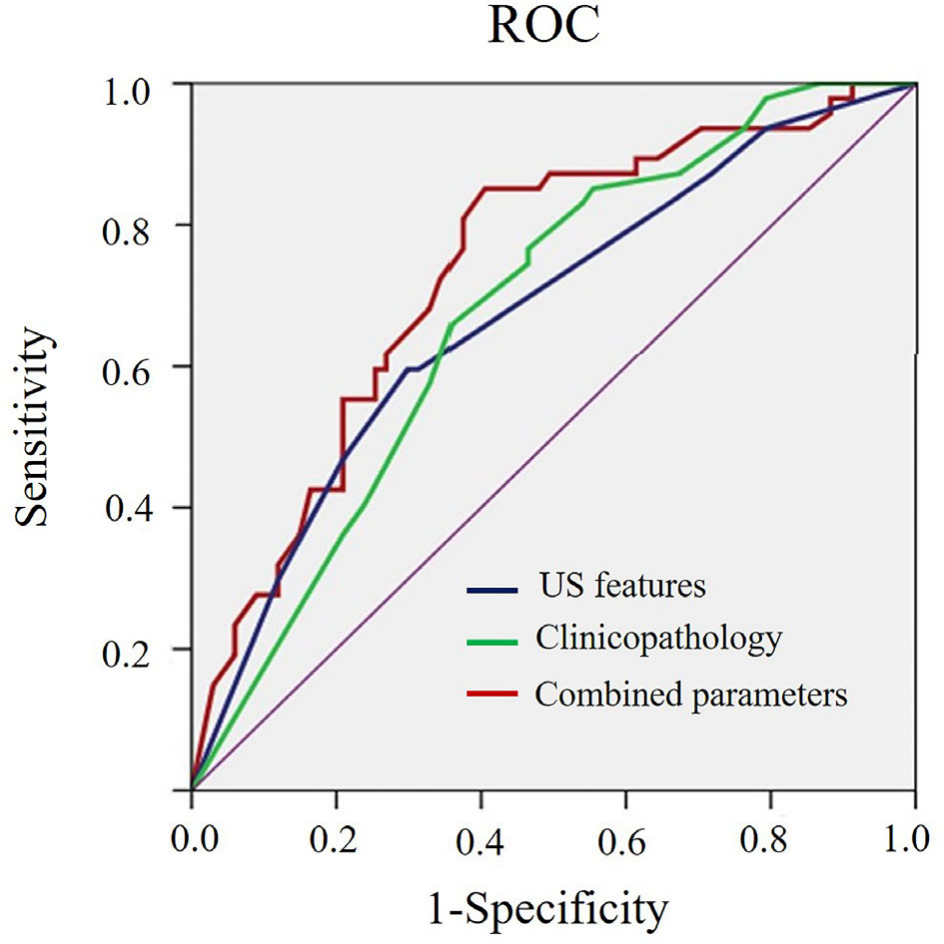
ROC curve for the prediction model of risk of recurrence based on sonographic features, clinicopathological characteristics, and the combined sonographic-clinical-pathological parameters of TNBCs.

As shown in **Table 3**, benign-like sonographic features correlated with high risk of recurrence (P<0.05). In contrast, malignant-like sonographic features correlated with low risk of tumor recurrence (P<0.05). **Table 4** shows that the fewer number of malignant sonographic features, the higher is the risk of recurrence. The tumor margin in the TNBC mass tended to be associated with the risk of recurrence (P=0.058). However, multivariate logistic regression analysis showed that there was no correlation between sonographic features and risk of tumor recurrence (P>0.05, **Table 5**). Regular shape, posterior acoustic enhancement, and the absence of calcification were “not” significantly associated with high risk of tumor recurrence (P=0.091, OR=2.04 for regular shape; P=0.166, OR=1.79 for posterior acoustic enhancement; and P=0.097, OR=2.21 for absence of calcification). To predict the risk of recurrence, the model based on sonographic features of regular shape, posterior acoustic enhancement, and absence of calcification were comparable (AUC=0.674, 95% CI 0.573–0.774) in the model based on the clinicopathological characteristics, such as tumor size, histological grade, Ki-67 expression, and axillary lymph node metastasis (LNM, AUC=0.678, 95% CI 0.580–0.775). The US-based and clinicopathology-based models showed equivalent results as seen by the AUC (P=0.956). As illustrated in **Table 6** and **Figure 5**, the combination of sonographic features and clinicopathological characteristics improved the prediction model (AUC=0.737, 95% CI 0.644–0.829). AUC was similar between the two types of models (combined model vs US-based model: P=0.083; combined model vs clinicopathology-based model: P=0.112).

**Table 3:** Association between sonographic features of TNBCs and risk of recurrence (Univariate Logistic regression analysis).

	High risk of recurrence	Low risk of recurrence	P value
**Shape**			0.014*
Regular	24 (51.1%)	19 (28.4%)	
Irregular	23 (48.9%)	48 (71.6%)	
**Margin**			0.058
Circumscribed	8 (17.0%)	4 (6.0%)	
Uncircumscribed	39 (83.0%)	63 (94.0%)	
**Posterior acoustic enhancement**			0.038*
Yes	23 (48.9%)	20 (29.9%)	
No	24 (51.1%)	47 (70.1%)	
**Calcification**			0.028*
Yes	8 (17.0%)	24 (35.8%)	
No	39 (83.0%)	43 (64.2%)	

*Indicates statistical significance.

**Table 4:** Association between the number of sonographic features with the risk of recurrence of TNBCs.

US feature group	Risk of recurrence group	
	Low risk	High risk
No malignant features	11 (35.5%)	20 (64.5%)
1–2 malignant features	42 (63.6%)	24 (36.4%)
3–4 malignant features	14 (82.4%)	3 (17.6%)
**P value**	**0.003**	

**Table 5:** Multivariate Logistic regression analysis for sonographic features associated with high risk of recurrence.

Sonographic features	Multivariate Analysis			
	B	SE	P value	OR (95%CI)
Regular shape	0.71	0.42	0.091	2.04 (0.89–4.66)
Posterior acoustic enhancement	0.58	0.42	0.166	1.79 (0.79–4.06)
Without calcification	0.79	0.48	0.097	2.21 (0.87–5.66)

B, regression coefficient; SE, standard error; OR, odds ratio; CI, confidence interval.

**Table 6:** Performance using sonographic features, clinicopathological characteristics and combined sonographic–clinical–pathological parameters to predict the high risk of recurrence of TNBCs.

Method	Sensitivity (%)	Specificity (%)	AUC (95%CI)	P value
US features	59.6	70.2	0.674 (0.573–0.774)	0.0006
Clinicopathology	76.6	53.7	0.678 (0.580–0.775)	0.0003
Combined	85.1	59.7	0.737 (0.644–0.829)	<0.0001

US, ultrasound; AUC, area under curve; CI, confidence interval.

Sonographic features include regular shape, posterior acoustic enhancement and without calcification; Clinicopathological characteristics include tumor size >2cm, histological grade III, Ki-67 ≥40% and presence of LNM.

## Discussion

TNBCs bear genomic, clinical, and imaging heterogeneity (2–4, 6, 8, 14–16). This study demonstrated the correlation between gene expression and sonographic features. Benign-like sonographic features correlated with the upregulated signature of HIF1A-AS2; while malignant-like sonographic features correlated with the downregulated signature of CHRDL1 in TNBCs. Sonographic features of TNBCs represent the risk of tumor recurrence that is based on the mRNA and lncRNA signatures. TNBCs with benign-like sonographic appearances have a higher risk of recurrence than those with malignant sonographic appearances.

CHRDL1 mRNA has been reported to have tumor suppressing effects on melanoma (17) and TNBC (2). lncRNAs cannot encode proteins, but they can regulate the expression of protein encoding genes involved in epigenetics, transcription, post-transcriptional modification, and translation. They also affect the occurrence, development, invasion, and metastasis of various tumors, such as breast cancer (18), bladder cancer (19), gastric cancer (20), colorectal cancer (21), and glioblastoma (22). The expression of HIF1A-AS2 is higher in TNBC than that in non-TNBC cancers and surrounding normal breast tissue (2, 18). HIF1A-AS2 improves the tolerance of tumor cells to hypoxic conditions, induces angiogenesis, and promotes growth and invasion of tumors by regulating the HIF-1α pathway (23). Jiang et al. suggested that HIF1A-AS2 affect the coding process by interrupting metabolism; AK124454 is involved in cell division and cell cycle (2). We have also previously reported the correlation between high expression of HIF1A-AS2 and AK124454 and increased risk of TNBC recurrence (2).

The integrated mRNA–lncRNA model predicted the risk of tumor recurrence reliably since it was based on 33 paired TNBC and normal tissues and a training set of 137 TNBCs with follow-up data on RFS. The reliability of the integrated model was further verified using 138 TNBC patients and 82 TNBC patients undergoing taxane-based neoadjuvant chemotherapy (2). However, to the best of our knowledge, this is the first report demonstrating that high risk of recurrence represents increased invasiveness that correlated with benign-like sonographic features.

In this study, sonographic features of TNBCs correlated with specific mRNAs and lncRNAs that are key regulators of TNBC cell proliferation. The sonographic features that were obtained using US rely on the acoustic characteristics of the tumor region that results from the interaction between tumor and normal tissues. The rate of TNBC tumor proliferation determines the possibility of interactions with the normal breast tissues. Enhanced cell proliferation allows specific cellular components to dominate in the tumor, thereby resulting in reduced fibrosis, infiltration, and reflection interferences (24, 25). Posterior acoustic enhancement results from the enhanced propagation of US energy owing to the lack of reflective interfaces. Upregulated RNAs that are involved in cell proliferation result in the occurrence of benign-like sonographic features, whereas downregulated RNAs reduce the rate of cell proliferation and result in the occurrence of malignant-like sonographic features. Our results are in accordance with this regulatory pattern. The extent of cell proliferation and differentiation is an important index for predicting tumor malignancy. Therefore, sonographic features of TNBCs may be an indirect indicator for tumor invasiveness. We have previously reported that sonogram features of breast cancers are reflective of tumor proliferation and invasiveness (26, 27). However, in this study, the effect of HIF1A-AS2 and CHRDL1 in determining the growth pattern of the tumor based on US was not studied in animals. Thus, exploring this in the future will enhance the utility of US as an important auxiliary technique to determine tumor characteristics and clinical outcomes in patients.

The sonographic difference between TNBCs and non-TNBCs was reported (28–30). However, the diversity in the sonographic features have not been studied. We have previously reported the variety of sonographic features of TNBCs and their correlation with clinicopathological and IHC characteristics (8). Understanding the biological basis for the variety in sonographic features is crucial in improving the reliability of diagnosing benign breast tumors by US specialists (8). We have demonstrated in this study that benign-like TNBCs correlate with higher risk of recurrence. US appearance of TNBCs can be used as an auxiliary parameter in addition to clinicopathological characteristics to stratify patients based on risk of relapse and develop personalized treatment in lieu of surgery.

However, this study has some limitations. First, the sample size of only 114 cases and the subpar quality of US imaging that were collected 6–8 years ago may affect the data interpretation. Second, we were not able to determine the correlation between sonographic features and RFS outcomes in this study; however, this is one of our ongoing projects. The last but not the least, there were only data on US images with no MRI data which may warrant further study.

## Conclusion

Sonographic features of TNBCs correlated with the risk of tumor recurrence that was predicted using the mRNA–lncRNA signatures. However, this correlation should be further verified using a larger patient cohort.

## Data Availability Statement

The raw data supporting the conclusions of this article will be made available by the authors, without undue reservation.

## Ethics Statement

The studies involving human participants were reviewed and approved by Institutional review board at Fudan University Shanghai Cancer Center. The patients/participants provided their written informed consent to participate in this study.

## Author Contributions

Conception and design: J-WL. Administrative support: S-CZ, CC. Provision of study materials or patients: CC. Collection and assembly of data: JZ, NL. Data analysis and interpretation: J-WL, Z-TS. Manuscript writing: all authors. Final approval of manuscript: all authors. All authors contributed to the article and approved the submitted version.

## Funding

This study was funded by National Natural Science Foundation of China (No. 81627804, 81830058), Shanghai Science and Technology Development Foundation of China (No. 18411967400), and Shanghai Anticancer Association SOAR Project (No. SACA-AX201905).

## Conflict of Interest

The authors declare that the research was conducted in the absence of any commercial or financial relationships that could be construed as a potential conflict of interest.
